# Individualized Nutritional Support for Hospitalized Patients With Oropharyngeal Dysphagia After Stroke: A Randomized Controlled Trial

**DOI:** 10.3389/fnut.2022.843945

**Published:** 2022-04-13

**Authors:** Xiu-Li Yan, Zhuo Liu, Ye Sun, Peng Zhang, Xue-Yan Lu, Fei Mu, Juan Du, Yi Yang, Zhen-Ni Guo

**Affiliations:** ^1^Department of Neurology, The First Hospital of Jilin University, Changchun, China; ^2^School of Nursing, The Fourth Military Medical University, Xi'an, China; ^3^Department of Neurology, Stroke Center & Clinical Trial and Research Center for Stroke, The First Hospital of Jilin University, Changchun, China; ^4^Reproductive Medical Center, Tangdu Hospital, The Fourth Military Medical University, Xi'an, China; ^5^Department of Pharmacy, Xijing Hospital, The Fourth Military Medical University, Xi'an, China

**Keywords:** stroke, oropharyngeal dysphagia, nutritional support, volume-viscosity swallow test, intermittent oroesophageal tube feeding

## Abstract

**Objectives:**

Post-stroke dysphagia may cause aspiration pneumonia, malnutrition, dehydration, and other complications. However, data on the effects of nutritional supplementation and its value after stroke are insufficient. We aimed to evaluate the effect of an individualized 1-week nutrition intervention program on swallowing function and nutritional status in stroke patients with oropharyngeal dysphagia.

**Methods:**

This study comprised the control group receiving oral nutritional support and continuous nasogastric tube feeding according to the results of the water swallow test (WST). The intervention group additionally underwent a volume-viscosity swallowing test (V-VST) and intermittent oroesophageal tube feeding based on WST. The outcomes were measured after 7 days of intervention, including the improvement of swallowing function assessment by WST, biochemical parameters, such as total serum protein, serum albumin, hemoglobin levels and body composition. This trial was registered with the Chinese Clinical Trial Registry, identifier ChiCTR 2100054054.

**Results:**

In total, 173 participants completed the study between September 1, 2020, and April 30, 2021. Patients receiving individualized nutritional support showed a more significant improvement in the total effective rate of swallowing function (95.3% vs. 85.1%, *P* < 0.05). After the intervention, the total serum protein level (0.97 ± 0.41 vs. −0.83 ± 0.47 g/L; *P* < 0.05), serum albumin level (0.33 ± 0.28 vs. −1.39 ± 0.36 g/L; *P* < 0.001) and lean tissue mass (0.13 ± 0.35 vs. −1.00 ± 0.40 g/L; *P* < 0.05) increased in the intervention group. The decrease of hemoglobin levels in the control group was more evident (−6.17 ± 1.63 vs. −0.64 ± 1.40 g/L; 95%CI, −9.78 to −1.28; *P* = 0.001). The difference of phase angle between the two groups was statistically significant (5.93 ± 0.88° vs. 5.77 ± 0.78°; *P* = 0.035), but not in body fat mass.

**Conclusions:**

In stroke patients with oropharyngeal dysphagia, the use of individualized nutritional support based on V-VST and intermittent oroesophageal tube feeding during the first week of hospitalization improved swallowing function and maintained nutritional status.

**Clinical Trial Registration:**

https://clinicaltrials.gov/, identifier: ChiCTR 2100054054.

## Introduction

Stroke has become the second leading cause of death worldwide (1). Oropharyngeal dysphagia (OD) is a common complication after stroke, with an incidence rate of OD within 3 days after stroke being 22–65% (2). The incidence rate of aspiration is >40% in patients with OD (3). OD can also cause a variety of other complications, including malnutrition, dehydration, and weight loss (4). In addition, the length of hospital stays and healthcare costs will increase with OD (5). Studies have shown that early detection of OD may reduce the risk of aspiration pneumonia and nutritional complications in stroke patients (6). However, the supporting evidence of nutritional support is insufficient, and there is growing interest about the effects of individualized nutritional support during the acute phase of stroke on swallowing function and nutritional status.

The diagnosis of OD requires clinical screening with the goal of quickly identifying patients at risk for OD who require clinical assessment. The 30-ml water swallowing test (WST) is a useful bedside screening tool for OD in stroke patients (7). In addition to clinical screening, the diagnosis of OD requires further clinical assessment. The volume-viscosity swallow test (V-VST) is a validated tool for the systematic assessment and clinical diagnosis of OD and provides accurate indications of the optimal bolus volume and viscosity for patients with OD. The diagnostic sensitivity and specificity of V-VST for OD can reach 93.17% and 81.39%, respectively (8). The design and implementation of texture-modified diet intervention based on V-VST results can effectively improve oral intake, weight, handgrip strength, and phase angle in older adults with OD (9).

Nutritional status may deteriorate during the first week after stroke, which is the first problem to be solved for stroke patients with OD. Studies have demonstrated that early nutritional support can improve the prognosis of patients at nutritional risk (10, 11). To facilitate recovery of swallowing function and improve the nutritional status of stroke patients, based on the two clinical screening and assessment tools mentioned above, we constructed an individualized nutritional management plan, provided graded nutrition management for stroke patients with OD according to the clinical assessment results, and made nurses play a leading role in the evaluation and intervention of swallowing disorders. The purpose of this study was to evaluate the effect of this individualized nutritional management plan on the swallowing function and nutritional status of stroke patients with OD, and hypothesize that it could improve the swallowing function of patients and maintain their nutritional status as much as possible.

## Materials and Methods

The study protocol was approved by the Ethics Commitment of the First Hospital of Jilin University (registration number 20K056-001), in accordance with the ethical recommendations of the Declaration of Helsinki, and registered at the Chinese Clinical Trial Registry (ChiCTR 2100054054). All participants were fully informed and written consent was obtained.

### Study Design and Participants

This study was a 7-day, single-center, randomized controlled, single-blinded, two-parallel group intervention study of post-stroke patients with OD. The study was conducted at the First Hospital of Jilin University, Changchun, China, between September 2020 and April 2021.

This study comprised consecutively admitted hospitalized stroke patients. According to the guidelines, all patients underwent formalized screening for OD by WST within 24 h after admission and before taking their first sip of water and food. Baseline evaluation was performed by registered nurses who were not co-investigators.

The inclusion criteria were as follows: (1) patients aged ≥ 18 years, (2) patients who met the diagnostic criteria of guidelines for the early management of patients with acute ischemic stroke (12) and underwent head computed tomography scanning or magnetic resonance imaging, (3) patients with Glasgow Coma Scale score ≥ 13 points, (4) patients with results of WST being II or above, and (5) patients with Nutrition Risk Screening 2002 score ≥ 3 points. The exclusion criteria were as follows: (1) patients with dysphagia before admission, (2) patients with significantly abnormal gastrointestinal function, frequent vomiting or diarrhea, or with enteral nutrition contraindications, (3) patients who had received enteral nutritional support before admission, (4) patients with poor compliance, who failed to complete follow-up, with incomplete clinical data, and with other conditions considered inappropriate for inclusion by researchers, and (5) patients and their immediate family members who did not want to be enrolled.

Patient characteristics were obtained through electronic medical records, which included the age, sex, comorbidity, National Institute of Health Stroke Scale (NIHSS) score, TOSTA criteria, length of stay, and hospitalization expenses.

### Randomization

The patients were randomly divided into an intervention group (*n* = 90) and a control group (*n* = 90) at a ratio of 1:1 using a computer-generated random number table. Random assignment with treatment allocation information was sealed in non-transparent envelopes by a research assistant who was not involved in patient assessment and intervention. All participants and investigators were aware of group assignment, but the outcome assessment was performed by masked nurses and medical technicians.

### Procedures

The multidisciplinary nutrition management team was composed of two neurology attending physicians, three neurology specialist nurses, a stroke health manager certified by the Stroke Prevention and Control Engineering Committee of the National Health Commission (13), a nutritional specialist and a rehabilitation therapist.

The attending physicians were responsible for clinical decisions and prescription of medical advice. Nurses were trained by rehabilitation therapists before performing the V-VST. The rehabilitation therapist was also responsible for swallowing therapy in both groups. The stroke health manager was responsible for providing advice to prevent malnutrition during hospitalization and to measure the human body composition of the two groups of patients. The dietitian worked with physicians and nurses to calculate adequate energy intake and guide the patient's diet.

### Control Group

**Figure 1:**
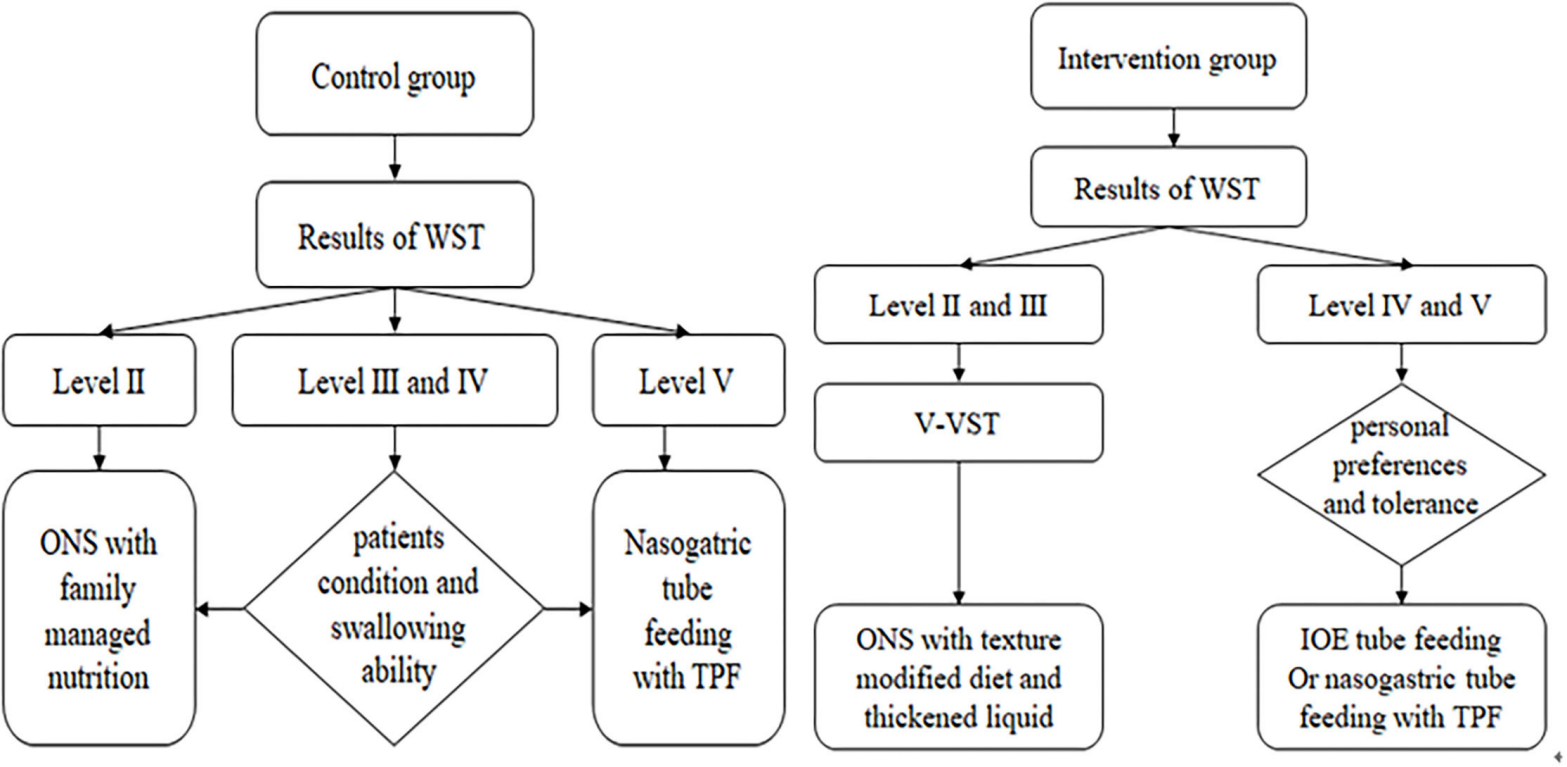
Implementation basis and method of nutritional support. WST, water swallow test; ONS, oral nutritional support; TPF, enteral nutritional suspension; V-VST, volume-viscosity swallowing test; IOE, intermittent oroesophageal.

According to the results of the WST of patients in the control group, an appropriate way of eating was selected. Patients with WST level II were recommended oral nutritional support (ONS) with family managed nutrition. With WST level III and IV, the rehabilitation therapist comprehensively considered the patient's condition and swallowing ability and selected ONS or continuous nasogastric tube feeding. The preparation of family managed nutrition followed the principles of easy digestion, low-salt, low-fat, and high-quality protein diet and selected semiliquid or liquid food texture. Patients with WST level V patients had indwelling nasogastric tubes as per medical advice (Figure 1).

Enteral nutritional suspension (TPF) produced by Nutricia Corp., which is a non-elemental enteral nutritional agent, was selected for enteral nutrition preparation. The caloric value of TPF was 100 kcal/100 ml, and the energy prescription recommendation was 25–30 kcal/kg/day according to the 2016 Society of Critical Care Medicine and American Society for Parenteral and Enteral Guidelines for the provision and assessment of nutritional support therapy in critically ill adult patient (14).

### Intervention Group

**Figure 2:**
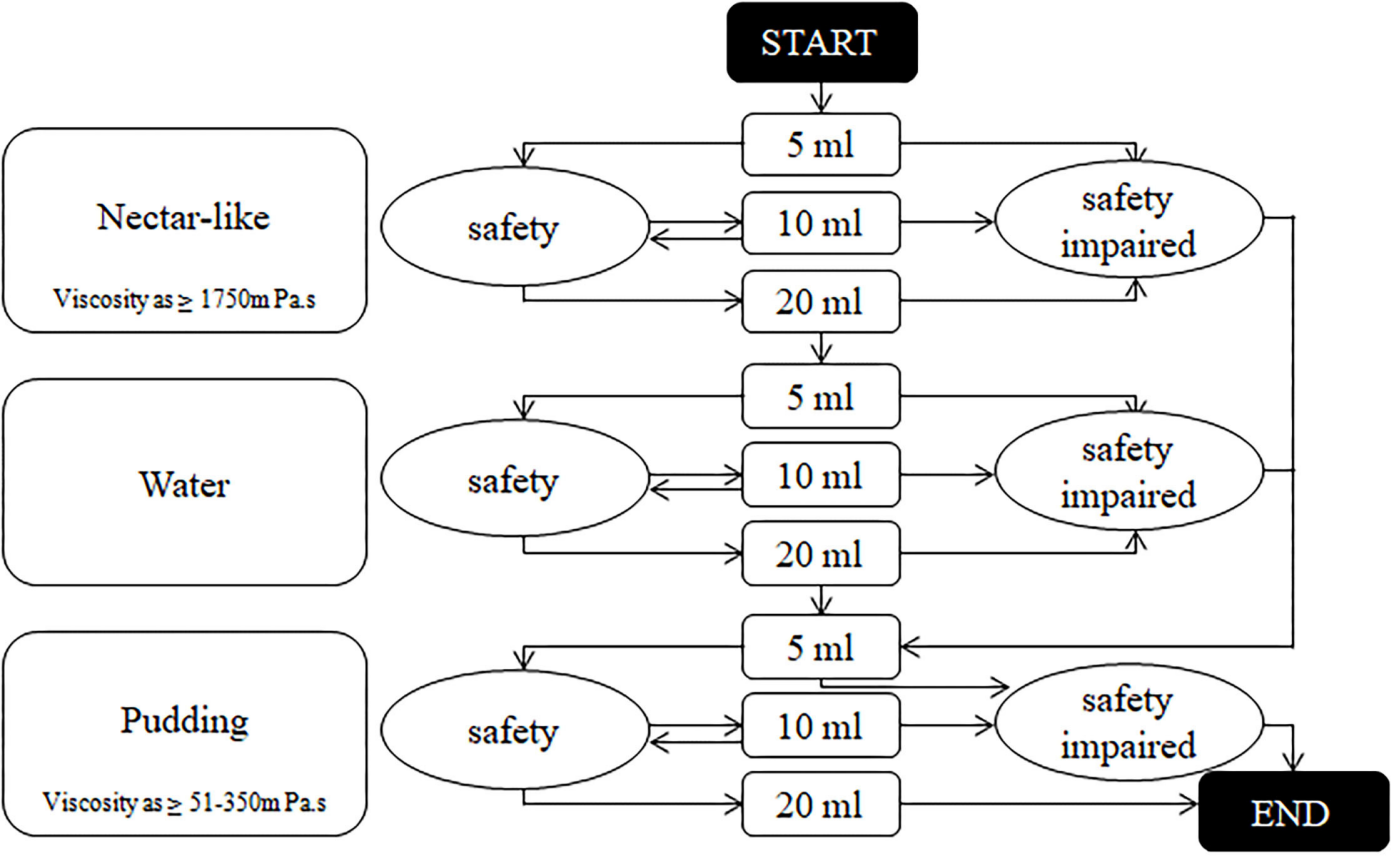
The algorithm of volume-viscosity swallowing test. Output result was the viscosity and sip size of the previous stage before any signs of safety impaired.

Patients with level II and III WST continued to use the V-VST for assessment (Figure 2). Different viscosities (nectar-like, pudding) were prepared using Resource Thicken Up (Nestle Company, Germany) and water. The volume of water was accurately measured using a syringe. The nectar-like viscosity was made up of 6.4 g Thicken Up and 140-ml water and the pudding viscosity was made up by adding 12.8 g Thicken Up to 140-ml water. Any signs of impaired safety (coughing, voice alterations, or a decrease in oxygen saturation of more than 3%) and impaired efficacy (labial seal, oral residue, pharyngeal residue, or repeated swallowing) were recorded during the assessment. The viscosity of the diet and liquid was determined according to the viscosity and volume tolerated in the V-VST.

For patients with level IV and V WST, intermittent oroesophageal (IOE) tube feeding and nasogastric tube feeding can be selected according to their personal preferences and tolerance. The enteral nutrition agents used in these two types of interventions were consistent with those in the control group. The caloric requirements were calculated by a dietitian using the Harris–Benedict estimation of basal energy expenditure. Daily protein intake was set at 1.2–1.5 g/kg. An individual nutritional plan was developed for each patient to reach these goals and caregivers of patients who underwent V-VST were taught to use thickeners to make diets with different viscosities according to the individual nutritional plan.

### Outcome Measures

The primary outcome was the assessment of swallowing function by nurses using the WST. The specific efficacy criteria were as follows: Recovery were swallowing dysfunction disappeared and the outcome of WST recovered to level I. Effective, dysphagia symptoms improved, and WST improved by a grade. Significantly effective were swallowing disorders significantly improved and WST improved by two grades or more. The degree of dysphagia remained the same as before the intervention with no improvement in the WST (15). The total effective rates were calculated using the following equation:


$$\begin{array}{l} \;Total\;efficacy\;rate\;=\;([number\;of\;recovery\;+\;number\;of\\ effective\;+\;number\;of\;significantly\;effective])/total\;number\;of\\ \;patients\;\times 100\%[-10pt] \end{array}$$

The secondary outcomes were biochemical parameters, including total serum protein, serum albumin and hemoglobin levels. Lean tissue mass, body fat mass and phase angle were measured by a stroke health manager using bioelectrical impedance analysis (BIA, Inbody S10, Biospace, Seoul, Korea). Measurements were performed before and after the intervention and in the supine position on an empty stomach in the morning. Phase angle was calculated using the following equation: Phase angle (°) = arctan (reactance/resistance) × (180/π). At the same time, we used unaffected side of limbs and trunk data for calculation to eliminate the effects of paralysis.

### Sample Size Calculation

The sample size was calculated according to the data of An et al. (16), and the results showed that the total effective rate of improvement in swallowing function was 66.0% after standardized nutritional intervention compared with 28.0% in the control group. Therefore, the individualized nutritional support was able to improve the proportion of improvement in swallowing function by 38.0%. In fact, considering the short duration of intervention, we estimate that the actual effect of individualized nutritional support may only reach 50% of the previous studies. In other words, we proposed that individualized nutritional support could increase the proportion of improvement in swallowing function by 19.0% at 7 days. Taking approximately 20% of dropout into account, a sample size of at least 90 participants per group was taken to reject the null hypothesis with a power of 0.9 and an alpha level of 0.05, which proves that the results of this study are effective.

### Statistical Analyses

Descriptive analysis of the general data and the measurement data in accordance with the normal distribution are expressed as the mean ± standard deviation (x ± s), and baseline comparisons between the two groups using independent sample *t*-tests and within-group changes were compared using paired *t*-tests. Measurement data that did not conform to a normal distribution were described by median and quartile range, together with the WST results, and the Mann-Whitney U test was used for comparison between the two groups. The counting data are expressed as a ratio of rate and composition and analyzed using the chi-squared tests or Fisher's exact test. Multiple linear regression model was used to examine the effectiveness of individualized nutritional support on nutritional status and body composition, adjusting for confounding factors like age, gender, comorbidities, and baseline value for each dependent variable. Statistical analyses were performed using IBM SPSS Statistics for Windows version 24.0. A two-tailed *P* value of < 0.05 indicated statistical significance.

## Results

### Demographic Information

**Figure 3:**
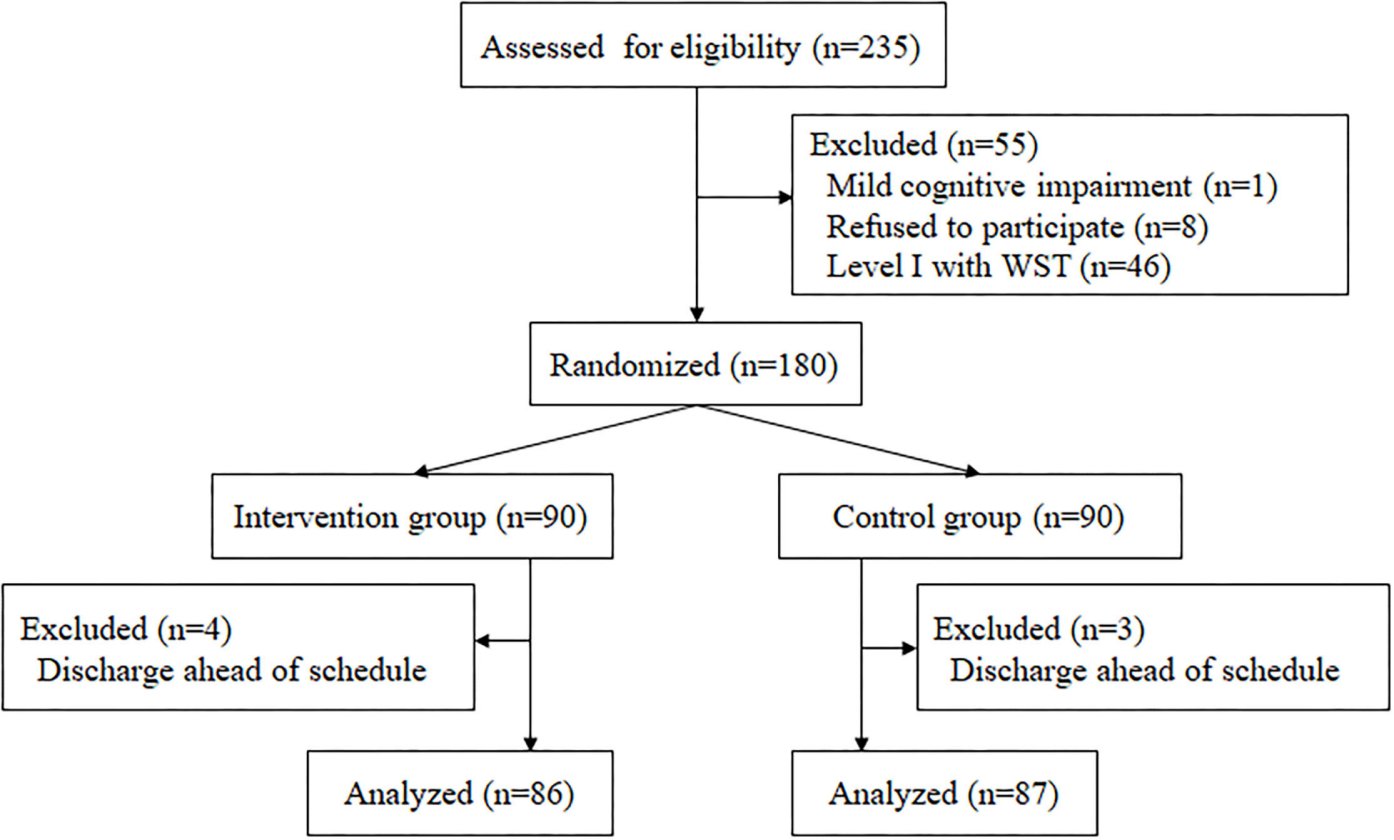
Flow chart of participants included in this study.

A total of 180 (90 in the intervention group and 90 in the control group) stroke patients with OD were included. During the study period, seven patients were excluded due to discharge ahead of schedule. Finally, 86 patients in the intervention group and 87 patients in the control group were analyzed (Figure 3). The baseline characteristics of the participants are presented in Table 1. There were no significant differences in age, sex ratio, medical history, smoking and drinking history, TOSTA criteria, and NIHSS scores between the two groups. The average length of stay and medical expenses did not differ significantly between the control and intervention groups.

**Table 1:** Baseline characteristics between control and intervention groups.

**Variables**	**Control group (*n* = 87)**	**Intervention group (*n* = 86)**	***t*/χ^2^**	***P* value**
Age (mean ± SD, years old)	61.79 ± 10.60	62.97 ± 10.60	−0.727	0.468
**Gender (n, %)**			1.113	0.292
Male	65 (74.7)	58 (67.4)		
Female	22 (25.3)	28 (32.6)		
**TOSTA criteria (n, %)**			1.230	0.894
Large artery atherosclerosis	36 (41.4)	33 (38.4)		
Cardioembolism	4 (4.6)	3 (3.5)		
Small-artery occlusion	2 (28.7)	31 (36.0)		
Other determined etiology	7 (8.1)	6 (7.0)		
Undetermined etiology	15 (17.2)	13 (15.1)		
**Comorbidities (n, %)**
Stroke	29 (33.3)	18 (20.9)	3.362	0.067
Hypertension	57 (65.5)	48 (55.8)	1.707	0.191
Diabetes	32 (36.8)	21 (24.4)	3.111	0.078
Coronary heart disease/Atrial fibrillation	19 (21.8)	16 (18.6)	0.280	0.596
Dyslipidemia	21 (24.1)	29 (33.7)	1.933	0.164
Smoking	38 (43.7)	35 (40.7)	0.158	0.691
Alcohol use	34 (39.1)	27 (31.4)	1.119	0.290
NIHSS, median (IQR)	3.0 (1.0, 6.0)	3.5 (1.0, 8.0)	−0.473	0.636
Length of stay (mean ± SD, day)	9.72 ± 2.03	9.97 ± 2.29	−0.733	0.465
Hospitalization expenses (mean ± SD, yuan)	30,225.11 ± 20840.69	25,294.17 ± 19498.21	1.607	0.110

*SD, standard deviation; NIHSS, National Institutes of Health Stroke Scale; IQR, interquartile range*.

### Improvement of Swallowing Function

**Figure 4:**
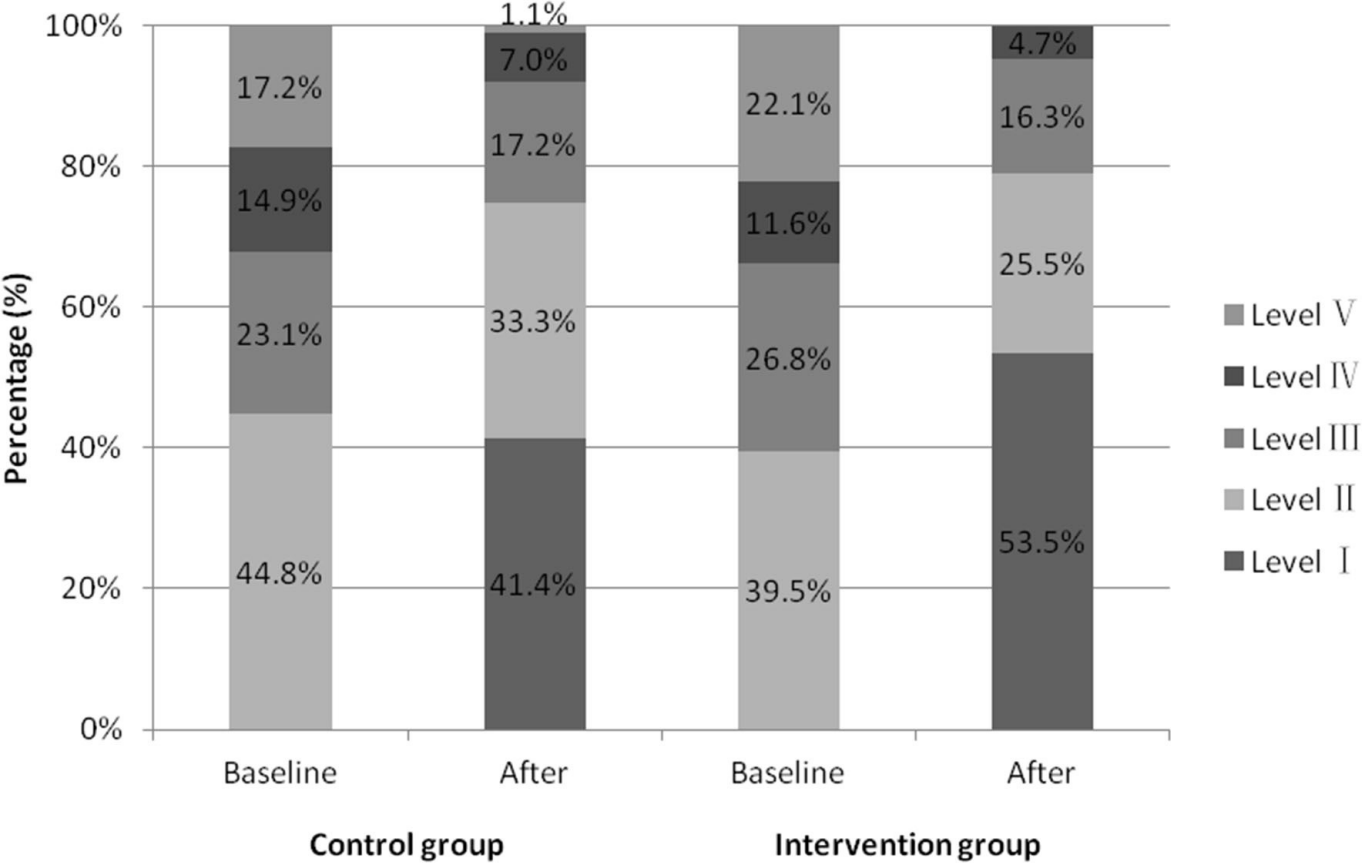
Proportion of water swallow test results before and after seven days of intervention in two groups.

In the control group, 63 participants were received ONS with family managed nutrition, and 24 were fed via nasogastric tube. Fifty seven participants in the intervention group received ONS with texture modified diet, 10 received IOE tube feeding, and 19 received nasogastric tube feeding. As shown in Figure 4, there was no significant difference in the outcomes of WST at baseline between the intervention and the control groups (*Z* = −1.455, *P* = 0.146). One week after baseline, the total effective rate of the intervention group (95.3%) was higher than that of the control group (85.1%), the difference was statistically significant (*P* <0.05; odds ratio, 0.278; 95% confidence interval [CI], 0.087–0.889; Table 2).

**Table 2:** Improvement of swallowing function in the two groups.

	**Recovery**	**Effective**	**Significantly effective**	**Total efficacy rate (%)**
Control group (*n* = 87)	36 (41.4%)	24 (27.6%)	14 (16.1%)	74 (85.1)
Intervention group (*n* = 86)	46 (53.5%)	14 (16.3%)	22 (25.6%)	82 (95.4)
χ^2^				5.169
*P*				0.023

### Biochemical Parameters and Body Composition

The baseline biochemical parameters in the control and intervention groups were similar (Table 3). After the intervention, an increase in total serum protein level was observed in the intervention group, but not in the control group (0.97 ± 0.41 vs. −0.83 ± 0.47 g/L; 95%CI, −3.01 to −0.58; *P* < 0.05). Compared with the control group, serum albumin level was higher in the intervention group (36.59 ± 3.09 vs. 38.29 ± 2.58 g/L; *P* < 0.001). Hemoglobin levels in the two groups decreased, but the decrease in the control group was more evident (−6.17 ± 1.63 vs. −0.64 ± 1.40 g/L; 95%CI, −9.78 to −1.28; *P* = 0.001) than that in the intervention group.

**Table 3:** Baseline values and changes in biochemical parameters and body composition after intervention in the two groups.

**Variables**		**Control group (*n* = 87)**	**Intervention group (*n* = 86)**	***t / F***	***P***
Total serum protein (g/L)	Baseline	66.21 ± 4.78	65.94 ± 5.24	0.348	0.729
	After	65.38 ± 4.82	66.91 ± 4.13	3.355	0.001^a^
	Δ	−0.83 ± 0.47	0.97 ± 0.41^*^	−2.907	0.004
Serum albumin (g/L)	Baseline	37.98 ± 2.50	37.96 ± 3.14	0.038	0.970
	After	36.59 ± 3.09	38.29 ± 2.58	5.000	<0.001^a^
	Δ	−1.39 ± 0.36^*^	0.33 ± 0.28	−3.806	<0.001
Hemoglobin (g/L)	Baseline	144.69 ± 12.97	143.22 ± 14.46	0.703	0.483
	After	138.52 ± 12.95	142.58 ± 12.04	2.747	0.007^a^
	Δ	−6.17 ± 1.63^*^	−0.64 ± 1.40	−2.569	0.001
Lean tissue mass (kg)	Baseline	23.96 ± 4.41	23.25 ± 4.22	1.088	0.278
	After	22.96 ± 3.18	23.38 ± 3.98	2.033	0.044^a^
	Δ	−1.00 ± 0.40^*^	0.13 ± 0.35	−2.143	0.034
Body fat mass (kg)	Baseline	21.82 ± 4.54	21.47 ± 5.86	0.439	0.661
	After	22.15 ± 4.82	21.68 ± 4.34	−0.613	0.541^a^
	Δ	0.34 ± 0.45	0.21 ± 0.44	0.200	0.842
Phase angle (°)	Baseline	5.96 ± 0.69	5.97 ± 0.90	−0.048	0.962
	After	5.77 ± 0.78	5.93 ± 0.88	2.130	0.035 ^a^
	Δ	−0.19 ± 0.36^*^	−0.05 ± 0.46	−2.370	0.019

*Change was presented as mean ± standard error of mean*.

a *Adjusted for baseline value, age, gender, comorbidities and NIHSS*.

^Δ^*Change from baseline to seven days*.

* *Significant change within the group*.

Lean tissue mass remained largely unchanged in the intervention group compared to baseline, but slightly decreased in the control group(0.13 ± 0.35 vs. −1.00 ± 0.40 g/L; 95%CI, −2.17 to −0.09; *P* < 0.05), but there was no difference in body fat mass between the two groups. Individualized nutritional support kept the phase angle basically stable in the intervention group, while the control group decreased. After we controlled for age, gender, comorbidities, disease severity, and baseline values, the difference between the two groups was statistically significant (5.93 ± 0.88° vs. 5.77 ± 0.78°; *P* = 0.035).

## Discussion

In this study, we found that individualized nutritional support within the first week of hospitalization with modified texture diet, thickened liquids, and IOE tube feeding in this study improved swallowing function and effectively maintained the nutritional status of post-stroke OD patients. The higher overall effective rate of swallowing disorder improvement in the intervention group may be due to the implementation of IOE tube feeding. During oral intubation, the posterior pharyngeal and swallowing reflexes and promoted the recovery of swallowing function. Studies have shown that compared with continuous tube feeding, IOE tube feeding could significantly increase the rate of dysphagia function improvement by 5.22 times, increase total serum protein and serum albumin levels of stroke patients with dysphagia, and was more conducive to the conversion of patients to complete oral feeding (17–19). According to the guidelines (20), we respect the wishes of patients and allow them to participate in treatment decision-making, which may help improve their cooperation and swallowing function. Sezgin et al.'s study also confirmed that fluid thickeners could improve swallowing functions (21).

Due to swallowing difficulties, patients with OD have reduced fluid and nutrient intake in both the acute and recovery stages of stroke (22). The European Society for Clinical Nutrition and Metabolism guidelines for neurology recommend screening for dysphagia in all patients with stroke before oral intake (23). As a tool for clinical screening of impaired swallowing safety and effectiveness in patients with dysphagia, V-VST can also direct healthcare personnel to better manage nutrition and reduce the risk of malnutrition (7). We performed V-VST for patients with WST at grade II and III in the intervention group, on the premise of ensuring safety and effectiveness as far as possible, selected the most appropriate food consistency and quantified the food volume for patients, reduced the chance of indwelling gastric tube for patients with swallowing disorders, and retained the normal eating experience of patients, which is conducive to the recovery of patients' diseases and improves their swallowing function, consistent with the results of Clavé et al. (24). The texture-modified diet and thickened liquids may increase the intake of patients in the intervention group. Therefore, compared with the family managed nutrition in the control group, the individualized nutrition management scheme better maintained the total serum protein, serum albumin and hemoglobin level.

The Academy of Nutrition and Diet and the American Society for Parenteral and Enteral Nutrition declared that loss of muscle is an important characteristic of malnutrition (25). After 7 days of intervention, the lean tissue mass of the control group decreased more, but the body fat mass between the two groups was not statistically significant, which may be due to the short intervention time. These two indicators will also be affected by the activity status after onset. The present study also used the Harris–Benedict equation to calculate the caloric requirement of patients, which was used by Otsuki et al. and improved the activities of daily living of older stroke patients in acute phase with malnutrition risk (26). The assessment of OD and individualized nutrition intervention in this study was performed mainly by nurses under the guidance and supervision of rehabilitation therapists and dietitians. Guo et al. also confirmed the role of nurses in a multidisciplinary team that improved the nutritional status of patients with dysphagia after stroke (27).

This study has some limitations. First, nutritional intervention was delivered for a relatively short period of time, and only the nutritional status and swallowing function of patients during hospitalization were observed. Second, the single-center and single-blinded design might have limited the general applicability of individualized nutritional support, which still needs to be examined. Third, the nutritional indicators used in this study may not objectively reflect the nutritional status of the patients. Follow-up investigations should be performed in future studies to observe the recovery of swallowing function and improvement of nutritional status and overall recovery after discharge. At the same time, whether the lean tissue mass and body fat mass can better reflect the nutritional status of stroke patients should be determined.

## Conclusion

On the premise of safety and effectiveness, the individualized nutrition management scheme ensures the eating experience of patients to the greatest extent, increases the participation of patients in the process of disease treatment, respects the subjective feelings of patients, and effectively improves the swallowing function and nutritional status of stroke patients with OD.

## Data Availability Statement

The original contributions presented in the study are included in the article/supplementary materials, further inquiries can be directed to the corresponding authors.

## Ethics Statement

The studies involving human participants were reviewed and approved by Ethics Commitment of The First Hospital of Jilin University. The patients/participants provided their written informed consent to participate in this study.

## Author Contributions

Z-NG, YY, and X-LY conceived and oversaw the study. ZL, YS, X-YL, FM, JD, and Z-NG performed data collection and critically revised the manuscript. ZL and PZ performed statistical analysis. X-LY and ZL wrote the manuscript. All authors contributed to the article and approved the submitted version.

## Funding

This work was supported by the Natural Science Foundation of Jilin Province, China to X-LY [grant number 20200201314JC], the Jilin Province Department of Finance to X-LY [grant number JLSWSRCZX2020-080], and the National Natural Science Foundation of China to FM [grant number 81903837].

## Conflict of Interest

The authors declare that the research was conducted in the absence of any commercial or financial relationships that could be construed as a potential conflict of interest.

## Publisher's Note

All claims expressed in this article are solely those of the authors and do not necessarily represent those of their affiliated organizations, or those of the publisher, the editors and the reviewers. Any product that may be evaluated in this article, or claim that may be made by its manufacturer, is not guaranteed or endorsed by the publisher.
